# Mr. Shaw Again

**Published:** 1911-03-18

**Authors:** 


### The Hospital

A Journal of

The Medical Sciences and Hospital Administration.

New Series. No. 215, Vol. VIII. [No. 1287, Vol. XLIX.] SATURDAY, MARCH 18, 1911

### Mr. Shaw Again.

Reflection on the experience of life can show many and many a set of events or characters which remind one's fancy of the Hegelian doctrine of the fusion of contradictories. How might a man of given disposition and training be expected to act in given circumstances? In one (specified) way, maintains very plausibly an able barrister: in just the opposite, his opponent establishes with an equally apparent cogency. The observer who has penetrated to the nursery thinks that women are by nature devoted and compassionate; while the philosopher in the ballroom notes mentally that fickleness and petty cruelty distinguish them most. To collect instances of this kind of thing is not an unprofitable line of thought at all. It does something towards eradicating prejudice and the tyranny of temperament, tends to free the intellect from its humiliating but frequent position of "valet to the emotions," disarms paradox of its power of disconcerting one; and, to come to a particular case, is a very useful—nay, an imperative—preparation to the perusal of Mr. Bernard Shaw's lately published homily * to the medical profession.

But in the matter of getting into Mr. Shaw's good graces, medical men are handicapped at the outset. No one of them who reads this book, disagreeing strongly with the first third of it, and approving of the rest, need fear for his own impartiality of mind. Doctors possess special knowledge, with the authority consequent upon special knowledge, whereas Mr. Shaw is a born revolutionary. Their work necessitates cutting flesh and bone, which is bound to revolt his humanitarian instincts. Leading clinicians find their most profitable patients among fashionable and monied people; and Mr. Shaw is anything but a courtier and hates plutocracy. Lastly, few things have put such a strain upon the economic resources of our present political system as the claims which visibly proceed from recent advances in the knowledge of preventive medicine. To seize on unfortunate discrepancies between precept and practice thus revealed has been repeatedly of late years Mr. Shaw's easy and (but not therefore) congenial task.

Thus the new Preface to his medical play of 1906 is, as might have been expected, hardly an eirenicon. As a matter of fact doctors are rated soundly throughout—although with the proffered consolation here and there that everybody else is nearly as bad—and always in the paradoxical, topsy-turvy manner alluded to in the opening paragraph of these remarks. The little recondite motive or characteristic which it requires Mr. Shaw's acute perception to define, and which happens to appeal to his temperamental convictions, is brought into the clearest prominence, while the great dominant one is not mentioned or explained away. There are men and women, says he, whom the operating-table seems to fascinate; half-alive people who, through vanity or hysteria, lose such feeble sense as they ever had of the value of their own organs and limbs. And so there are—but a hugely greater number (witness the vogue of the "bloodless surgeon") who care a great deal for their skin, and think many times before consenting to anything which threatens its continuity. Take, again, the opening remarks on vivisection. The successful rulers of history, he says, have succeeded by fascinating and intimidating their peoples by monstrous cruelties: similarly vivisection helps the doctor to rule "us." Well, certainly a little ostentation may sometimes be seen in those departments of medicine which try the nerves of a lay onlooker, but it is surely confined to the new dresser proud of his blood-stained overall, or the student who holds the rabbit's ear up for his teacher to inject. Of course, the fact of the matter is that the writer's objection to any rule at all of "us" is so great that his always keen vision becomes sharpened to the highest degree. Then, later, it is allowed that the divine thirst for knowledge is a greater motive. But if you cannot attain to knowledge without torturing a dog you must do without knowledge; besides (what ignorance of the colossal difficulties of physiological research to put it on the level, say, of re-wording a sentence!) a little resource would soon reveal other methods; and then the perfect type of the average vivisectionist's experiment is Charles Lamb's

Chinaman burning down a house to get roast pork.

But if one once loses sight of Mr. Shaw's peculiar fitness for the position of devil's advocate in this case, medical criticism of the Preface to "The Doctor's Dilemma" resolves itself into a mere flat enumeration of fallacies. He has a good deal of knowledge of medical subjects, too, both observed and otherwise acquired. Quite true it is that a doctor's reputation is at the mercy of the nurse; very small experience of practice is needed to show that in serious illness the patient's friends generally ask the nurse if she thinks the doctor "really understands the case," and they are much influenced by her opinion and the manner in which she gives it. And then (really, Mr. Shaw's habit of putting the small thing first, and the important one second, is infectious) this author has been a pioneer in pointing out the evils involved in "club-practice" amongst the poor, and the need for extension of the system of State-paid medical inspection. But it is necessary to say clearly that the tone of this treatise is altogether too drastic; medical men have no difficulty in keeping countenance when in each other's company; there is, as already hinted, a good deal less wrong with them than with those belonging to sister professions, and they decline with small thanks Mr. Shaw's proposal for lay arbitration when the question of operation comes up. It is necessary, because now only unenlightened or old-fashioned people fail to take this author seriously, and he is widely read among the leaders of the working classes, who do not lack for self-confidence, and are coming to have such a large say in all medico-sociological affairs. The style of the Preface is admirably strong and trenchant, as usual; but it is to be noted with disappointment that there are no new phrases. Mr. Shaw used to be so clever at these. Here one finds nothing except an obvious failure—"breath-bereaving brazenness"—which is reminiscent (no deadlier insult could be hurled!) of a piece of Elizabethan bombast.

* *The Doctor's Dilemma, Getting Married,* and *The Showing-up of Blanco Posnet.* By Bernard Shaw. London: Constable and Co. 6s.

